# Antioxidative, Antibacterial and Antiproliferative Properties of Honey Types from the Western Balkans

**DOI:** 10.3390/antiox11061120

**Published:** 2022-06-06

**Authors:** Marijana Sakač, Pavle Jovanov, Aleksandar Marić, Dragana Četojević-Simin, Aleksandra Novaković, Dragana Plavšić, Dubravka Škrobot, Renata Kovač

**Affiliations:** 1 Institute of Food Technology in Novi Sad, University of Novi Sad, Bulevar cara Lazara 1, 21000 Novi Sad, Serbia; marijana.sakac@fins.uns.ac.rs (M.S.); pavle.jovanov@fins.uns.ac.rs (P.J.); aleksandra.novakovic@fins.uns.ac.rs (A.N.); dragana.plavsic@fins.uns.ac.rs (D.P.); dubravka.skrobot@fins.uns.ac.rs (D.Š.); renata.kovac@fins.uns.ac.rs (R.K.); 2 Oncology Institute of Vojvodina, Put doktora Goldmana 4, 21204 Sremska Kamenica, Serbia; dcetojevicsimin@singidunum.ac.rs; 3 Department of Pharmacy, Singidunum University, Danijelova 32, 11000 Belgrade, Serbia

**Keywords:** honey, antioxidant activity, antibacterial activity, antiproliferative activity

## Abstract

This paper presents the physicochemical characteristics and antioxidative, antibacterial and antiproliferative effects of nineteen samples of different honey types (acacia, linden, heather, sunflower, phacelia, basil, anise, sage, chestnut, hawthorn, lavender and meadow) collected from different locations in the Western Balkans (Republic of Serbia, Kosovo, Bosnia and Herzegovina, and Northern Macedonia). Physicochemical parameters (moisture, pH, electrical conductivity, free acidity, and hydroxymethylfurfural [HMF]) were analysed. Based on the obtained results, all tested honey samples were in agreement with EU regulation. The antioxidant potential of honey samples was assessed by determination of total phenolic content (TPC) and evaluation of scavenging activity towards diphenilpicrylhydrazyl radicals (DPPH·). The highest phenolic content was found in basil honey (101 ± 2.72 mg GAE/100 g), while the lowest was registered in rapeseed honey (11.5 ± 0.70 mg GAE/100 g). Heather, anise, phacelia, sage, chestnut and lavender honey samples were also rich in TP, containing 80–100 mg GAE/100 g. DPPH scavenging activity varied among the samples being the highest for lavender honey (IC_50_ = 88.2 ± 2.11 mg/mL) and the lowest for rapeseed honey (IC_50_ = 646 ± 8.72 mg/mL). Antibacterial activity was estimated in vitro using agar diffusion tests and measuring minimal inhibitory concentration (MIC). Among investigated bacterial strains following resistant potencies were determined: *Escherichia coli* > *Escherichia coli* ATCC 8739 > *Enterococcus faecalis* > *Proteus mirabilis* > *Staphylococcus aureus* > *Staphylococcus epidermidis*. The linden honey from Fruška Gora (MIC values of 3.12% and 6.25% against *Staphylococcus aureus* and *Staphylococcus epidermidis*, respectively) and phacelia honey (MIC values of 6.25% and 3.12% against *S.* *Staphylococcus aureus* and *Staphylococcus epidermidis*, respectively) showed the strongest antibacterial activity. Antiproliferative activity was evaluated using the colorimetric sulforhodamine B (SRB) assay. The highest antiproliferative activity was obtained from linden honey sample 1 (IC_50_^MCF7^ = 7.46 ± 1.18 mg/mL and IC_50_^HeLa^ =12.4 ± 2.00 mg/mL) and meadow sample 2 (IC_50_^MCF7^ = 12.0 ± 0.57 mg/mL, IC_50_^HeLa^ = 16.9 ± 1.54 mg/mL and IC_50_^HT−29^ = 23.7 ± 1.33 mg/mL) towards breast (MCF7), cervix (HeLa) and colon (HT-29) cancer cells. Active components other than sugars contributed to cell growth activity.

## 1. Introduction

Honey is a natural sweetener produced by honeybees using nectar. It has been used not only for food, but also for therapeutic purposes.

The nutritional profile of honey is defined by the content of its main constituents, carbohydrates and water, as well as numerous minor compounds such as organic acids, proteins, amino acids, minerals, vitamins and other compounds [1]. It is mostly influenced by the type of nectar, secretions of flowering plants and/or excretions of plant-sucking insects, as well as climate conditions and soil composition [2].

In addition to its dietary effects, honey is known for health properties arising from its antioxidant nature, which is primarily provided by its phenolic composition, rather than the presence of ascorbic acid, carotenoid-like substances, organic acids, Maillard reaction products, amino acids and proteins [3]. The antioxidant properties of honey vary due to botanical and geographical variations.

The main polyphenols in honey are flavonoids, but phenolic acids are also present [4]. Phenolic compounds in honey depend on the type of nectar and therefore can serve as floral markers. Their presence in honey is associated with its medicinal properties. For example, antitumor effect of coriander honey was established using Ehrlich ascites carcinoma (EAC) model with mice and attributed to its antioxidant activity/polyphenol presence [5]. A list of beneficial roles of honey polyphenols against some human degenerative diseases was presented in the paper of Hossen et al. [6]. However, overall honey therapeutic properties imply not only its antioxidant nature, but antibacterial, bacteriostatic, anti-inflammatory, antimutagenic and other activities [7].

The antibacterial activity of honey was attributed to the high osmolarity and acidity of honey [8], as well as the presence of hydrogen peroxide, which is generated by glucose oxidase-mediated conversion of glucose in honey [9]. Additionally, phenolic compounds are responsible for the antimicrobial activity of honey [10]. Methyl syringate was evidenced to provide honey with its ability to scavenge superoxide free radicals and thus exerts its antibacterial activity [11].

Several studies demonstrate the anticancer activity of honey, i.e. raw honey shows a chemopreventive effect against various cancer cell lines and tissues in in vitro and in vivo studies [12]. This activity can be explained by different mechanisms including cell cycle arrest, induction of apoptosis, modulation of oxidative stress and immuno-modulation. Acacia honey inhibited the growth of human breast adenocarcinoma cells in a dose- and time-dependent manner by apoptotic cell death [13], while buckwheat honey was proven to express antiproliferative effect in in vitro study [14]. These evidences suggest the potential application of honey (or its active components) as a part of an alternative medical treatment of human tumours [15].

The Republic of Serbia is known to produce acacia, sunflower and linden honey as the most frequently used monofloral honey types [2,16], but some others are also present on the Serbian market (rapeseed and phacelia) [17].

Starting from the considered therapeutic properties of honey, the aim of this paper was to evaluate the antioxidative, antibacterial and antiproliferative effects of nineteen samples of different honey types (acacia, linden, heather, sunflower, phacelia, basil, anise, sage, chestnut, hawthorn, buckwheat, lavender and meadow) collected from different locations in the Western Balkans (Republic of Serbia, Kosovo, Bosnia and Herzegovina, and Northern Macedonia). Physicochemical parameters (moisture, pH, electrical conductivity, free acidity, and HMF) were also determined to ensure that honey samples fulfilled the requirements for honey quality [18].

## 2. Material and Methods

### 2.1. Honey Samples

Nineteen samples of different honey types (acacia, linden, heather, rapeseed, sunflower, phacelia, basil, anise, sage, chestnut, hawthorn, lavender and meadow) were obtained from beekeepers who declared their botanical origin. The harvesting period was 2019 and the samples were from different locations in the Republic of Serbia, Kosovo, Bosnia and Herzegovina, and Northern Macedonia (Table 1). The samples were packed in glass vessels and stored at room temperature (22 ± 1 °C) in a dark place until analyses.

### 2.2. Physicochemical Parameters

The physicochemical parameters of honey samples (moisture, pH, electrical conductivity and free acidity) were determined according to the methods of AOAC [19] and the Harmonised Methods of the International Honey Commission [20].

### 2.3. Hydroxymethylfurfural (HMF) Analysis

Sample preparation: The extraction procedure was described by Sakač et al. [2] based on the method of Rufián–Henares and De La Cueva [21] with some modifications made by Petisca et al. [22].

HPLC-DAD analysis: the HPLC method described by Ariffin et al. [23] and Tomasini et al. [24] was used to quantify HMF in honey samples. HPLC analysis was performed using a liquid chromatograph (Agilent 1200 series, Agilent Technologies Santa Clara, CA, USA) with a DAD detector and an Eclipse XDB-C18, 1.8 μm, 4.6 × 50 mm column (Agilent). The column temperature was 30 °C. The injection volume was 2 μL. The mobile phase consisted of two eluents, H_2_O (0.1% HCOOH) (A) and methanol (B). The flow rate was 0.75 mL/min. The isocratic elution was applied with the ratio A:B (90:10, *v*/*v*). The total run time was 5 min.

### 2.4. Total Phenolic Content

The Folin–Ciocalteu method described by Ferreira et al. [25] was used to determine total phenolic content (TPC) with some modifications. Honey sample (1 g) was dissolved in 20 mL of distilled H_2_O. The honey solution was used (8 mL) and mixed with 500 μL of diluted Folin–Ciocalteu reagents (1:2) for 3 min. Thereafter, 1.5 mL of sodium carbonate (25%) was added. The mixture was shaken and left to stand in the dark at 22 ± 1 °C for 2 h. The absorbance was measured at 750 nm using a spectrophotometer (Specord M40, Carl Zeiss, Jena, Germany). Gallic acid (1.25–31.25 μg/mL) was used as the standard for the construction of the calibration curve, and the TPC was expressed as gallic acid equivalents (GAE) (mg GAE/100 g of honey).

### 2.5. DPPH Radical Scavenging Activity

The ability of honey samples to scavenge 1,1-diphenyl-2-picrylhydrazyl radicals (DPPH·) was estimated using the method described by Hatano et al. [26], with some modifications.

A honey sample (2 g) was dissolved in 10 mL of distilled water, centrifuged (3000× *g*) and filtered. Then, 0.1 mL of each of the various concentrations of the honey solution (25.0, 50.0, 100, 200, 400, and 800 mg/mL) was diluted in 2.9 mL of methanol, and 1 mL of 90 μmol/L methanol solution of DPPH was added. The control was prepared using distilled water instead of honey solution. The reaction mixtures were vortexed and left to stand in the dark at 22 ± 1 °C for 1 h. The absorbance was measured at 517 nm using a spectrophotometer (Specord M40, Carl Zeiss, Jena, Germany). The IC_50_ value (mg/mL) was defined as the concentration of an antioxidant which was required to quench 50% of the initial amount of DPPH·.

### 2.6. Antibacterial Activity

Honey solutions were prepared by dissolving honey in sterile distilled water immediately prior to analysis to obtain a range of dilutions (25.0%, 12.5%, 6.25%, 3.125%, 1.56%, and 0.75%).

The antibacterial activity was tested against the gram-negative bacteria *Escherichia coli* (ATCC 8739), *Escherichia coli* I (clinical strain), and *Proteus mirabilis* (clinical strain), and gram-positive bacteria *Staphylococcus aureus* (ATCC 25923), *Staphylococcus epidermidis* (clinical strain), and *Enterococcus faecalis* (ATCC 29212).

The minimal inhibitory concentration (MIC) was determined by modified microdilution analysis [27]. Pure bacterial strains were subcultured on nutrient agar slants at 37 °C for 24 h, while suspensions of the tested strains corresponded to the McFarland 0.5 optical density ≈ 1.5 × 10^8^ CFU/mL. The MIC of the samples was determined following the addition of 10 μL of 2,3,5-triphenyl tetrazolium chloride (1% solution) and incubation at 37 °C for 2 h, until the development of the red colour. The lowest concentration of honey that inhibited bacterial growth, which was identified by the absence of red formazan, was considered as the MIC.

### 2.7. Antiproliferative Activity

The ATCC (American Type Culture Collection, Manassas, VA, USA) human tumor cell lines HeLa (cervcal carcinoma), MCF7 (breast epithelial adenocarcinoma), HT-29 (colon adenocarcinoma) and MRC-5 (normal fetal lung fibroblasts) were used for the estimation of cell growth activity. Cell lines were grown in Dulbecco’s modified Eagle’s medium (DMEM; PAA Laboratories GmbH, Pashing, Austria) with 4.5% glucose, supplemented with 10% heat-inactivated fetal calf serum (FCS; PAA Laboratories GmbH, Pashing, Austria), 100 IU/mL of penicillin and 100 µg/mL of streptomycin (Galenika, Belgrade, Serbia). Cell lines were cultured in 25 mL flasks (Corning, New York, NY, USA) at 37 °C in an atmosphere of 5% CO_2_, high humidity and sub-cultured twice a week. Single cell suspension was obtained using 0.1% trypsin (Serva, Heidelberg, Germany) with 0.04% EDTA.

Honey samples and standard (glucose) were dissolved and diluted in 0.9% NaCl to obtain a range of ten concentrations (0.15–50 mg/mL). All samples were filtered through a 0.22 μm Millipore (Millex-GV) membrane filters to obtain sterility.

Cell lines were harvested and plated into 96-well microtiter plates (Sarstedt, Newton, NC, USA) at a seeding density of 4–8 × 10^3^ cells per well, with a volume of 180 µL, and pre-incubated in medium supplemented with 5% FCS at 37 °C for 24 h. Serial dilutions of samples and solvent (20 µL per well) were added to test and control wells, respectively. Microplates were then incubated at 37 °C for the additional 48 h.

Cell growth was evaluated using the colorimetric sulforhodamine B (SRB) assay according to Skehan et al. [28], modified by Četojević–Simin et al. [29]. Effects on cell growth were expressed as a percent of the control and calculated as % Control = (A_t_/A_c_)·100 (%), where A_t_ is the absorbance of the test sample and A_c_ is the absorbance of the control. Corresponding dose-effect curves were drawn using Origin software (Version 8.0) and IC_50_ values (concentration of sample that inhibits cell growth by 50%) were determined. Results were expressed as mean ± SD of four measurements (*n* = 4 test samples and standard) and eight measurements (*n* = 8; controls).

### 2.8. Statistical Analyses

The data were processed statistically using the software package STATISTICA 10.0 (StatSoft Inc., Tulsa, OK, USA) and XLSTAT 2022.1 (Addinsoft, New York, NY, USA). Results were expressed as mean ± standard deviation of triplicate analyses for all measurements, except in the case of in vitro cell growth activity determination, which was performed in 4 or 8 repetitions. Furthermore, principal component analysis (PCA) was performed to graphically visualize the relationships between all analysed parameters and samples.

## 3. Results and Discussion

### 3.1. Physicochemical Characterisation of Honey

The physicochemical parameters of honey represent the useful indicators of its quality, which is necessary to be in accordance with EU regulation [18]. Also, these parameters can help in estimation of honey botanical origin [30]. Among physicochemical parameters, moisture content, electrical conductivity, pH, free acidity and HMF were determined and presented in Table 2.

The moisture content of honey depends on its botanical and geographical origin, as well as beekeeping/processing techniques and storage conditions [1,16]. The moisture content of examined honey samples was in the range of 14.9 ± 0.15 to 19.4 ± 0.15%, being the highest in rapeseed honey sample 1 and the lowest in meadow honey sample 1 (Table 2). All honey samples met the criterion for moisture content (max 20%) defined by the Codex Alimentarius Commission [18].

Free acidity results from the presence of H donors in honey, i.e. the presence of organic acids in equilibrium with their corresponding lactones and some inorganic ions. The fermentation of sugars into organic acids leads to incrased honey acidity. This parameter is limited to 50.00 meq/kg [18], and all the examined samples fulfilled this requirement (Table 2).

The pH limit is not deffined by Codex Alimentarius Commission [18], but it is recommended to be low in order to suppress microbiological growth. The pH values in the examined honey samples were between 3.36 ± 0.03 and 4.72 ± 0.02, being comparable with those presented in other papers [12,31,32]. This parameter has great importance during the extraction and honey storage, because it influences the texture, stability and shelf life of honey [33].

The electrical conductivity of honey samples ranged from 114 to 1251 μS/cm (Table 2). Acacia honey was characterised by the lowest conductivity (114 ± 2.65 μS/cm), which is in line with previously reported results for acacia and other light-coloured honeys [34]. Contrarily, dark honey types have higher conductivity levels [35], especially chestnut honey (1251 ± 41.7 μS/cm) (Table 2), whose conductivity correlates with its high mineral content [34,36]. In both cases (acacia and chestnut honey) electrical conductivity may be the marker of botanical origin. Regarding conductivity levels, all the samples were compliant with the level defined by the regulation [18] except heather and chestnut honey samples which are known to have conductivity values above 0.8 mS/cm [31] and, therefore, represent the exceptions in honey regulative [18]. The data are consistent with previously reported values of honey types from Serbia [16,17] and other European countries [32].

HMF is a marker of honey freshness. This non-enzymatic Maillard reaction product is acceptable at the values below 40 mg/kg for honey originating from non-tropical regions [18]. It is known that the amount of 10 mg/kg HMF naturally presents in honey [35]. Therefore, examined honey samples were considered fresh having HMF content from 1.81 ± 0.58 mg/kg (meadow 3) to 9.41 ± 0.70 mg/kg (sunflower) (Table 2).

### 3.2. Antioxidative Potential of Honey

The main antioxidants present in honey are polyphenols [31], which also exhibit bactericidal, anti-inflammatory, anti-allergenic, anticoagulant, and anti-cancer effects [37].

Differences in TPCs between examined honey samples were statistically significant (*p* ≤ 0.05) (Table 3) and arise from differences in honey botanical and geographical origin [1].

The highest phenolic content was found in the basil honey sample (101 ± 2.72 mg GAE/100 g), while the lowest was registered in the rapeseed honey sample 1 (11.5 ± 0.70 mg GAE/100 g) (Table 3). Similar TPC levels were reported in the study of Liu et al. [38] who determined TPC in honey ranging from 0.307 ± 0.01 to 0.822 ± 0.03 mg GAE/g or in the study of Can et al. [34] who found TPC levels between 16.02 and 120.04 mg GAE/100 g. Acacia honey samples 1 and 2 that belong to the light honey types were poor in polyphenols as reported in the paper of Marić et al. [39] for acacia honey from Serbia region. Among darker honeys heather honey samples 1 and 2 had high TPC (79.3 ± 1.01 mg GAE/100 g and 84.0 ± 3.83 mg GAE/100 g, respectively) being in line with findings of Alves et al. [31] and Can et al. [34] for heather honey. Anise honey was characterised by high TP content (98.7 ± 0.90 mg GAE/100 g) and this result is comparable with the finding of Gül and Pehlivan [40] (113.22 ± 0.46 mg GAE/100 g). Phacelia and sage honeys were also rich in polyphenols (Table 3). Chestnut honey had slightly less TP (88.8 ± 1.55 mg GAE/100 g) (Table 3) compared to the result of Can et al. (98.26 ± 17.77 mg GAE/100 g) [34] and Kaygusuz et al. (52.4–105.0 mg GAE/100 g) [41] for Turkish chestnut honey but despite its lower content, it is classified as honey rich in polyphenols as found in the paper written by Can et al. [34]. Lavender honey from Fruška Gora was superior in TPC (95.6 ± 1.06 mg GAE/100 g) compared to TPC found by Can et al. (53.39 ± 23.34 mg GAE/100 g) [34]. Rapeseed honey samples 1 and 2 represented honey types poor in polyphenols. TPC in rape honey from Poland was slightly higher—18.3 ± 3.61 mg GAE/100 g [42]. Although Piljac-Žegarac et al. [43] cited that heterofloral honeys from Croatia exhibited the highest mean TP content (58.75 mg GAE/100) among twenty-six honey samples of different floral origin (11 monofloral, 7 heterofloral, 8 special), our meadow honey samples 1–3 were much lower in phenolics (16.8 ± 0.50 to 26.5 ± 0.76 mg GAE/100 g), as already established in the paper written by Marić et al. [39]. Alves et al. [31] also found that multifloral honeys are light-colored and have low phenolic contents.

The antioxidant activity of honey samples was evaluated in DPPH assay and differed significantly among the samples (Table 3). The scavenging activity of different honey types on DPPH radicals expressed as IC_50_ value was in the range from 88.2 ± 2.11 mg/mL (lavender honey) to 646 ± 8.72 mg/mL (rapeseed honey sample 1) (Table 3). Linden honey sample 1 (from Fruška Gora) was found to be very potent (IC_50_ = 115 ± 9.00 mg/mL), as well as heather honey sample 1 (IC_50_ = 137 ± 7.55 mg/mL), while acacia honey samples exhibited low antioxidant activity (IC_50_ around 400 mg/mL). The lowest antioxidant activity was recorded for rapeseed honeys (IC_50_ around 640 mg/mL). Although the comparison of the obtained results with the data published by other authors is difficult due to the differences in expression of the antioxidant activity on DPPH radicals (% inhibition, IC_50_ values or some other), our finding regarding the heather honey samples potential is similar to that of Kuś et al. [42], who underlined the high antioxidant activity of heather honey. Contrary, acacia honey showed low DPPH activity (Table 3) as previously reported by Gül and Pehlivan [40]. Wilczyńska [44] established that acacia honey was the lowest potent honey type in terms of antioxidant activity on DPPH radicals, followed by goldenrods < rape < lime < nectar-honeydew < multifloral < buckwheat < honeydew < phacelia < heather. Our DPPH results are in line with the mention order except rape honey samples 1 and 2, which were the lowest in DPPH activity.

The results of honey DPPH activity were correlated with TP contents of honey samples (R^2^ = −0.816) indicating that antioxidant activity is primarily a consequence of the presence of polyphenolics that has been noticed before by other authors [40,42].

### 3.3. Antibacterial Activity

The antibacterial properties of honey could be attributed to the individual or synergetic effects of the honey acidity, osmolality, the presence of enzymatically generated hydrogen peroxide and the presence of polyphenols [45,46] or some other compounds (e.g., methylglyoxal in manuka honey) [27].

Antibacterial activity against the gram-negative bacteria: *Escherichia coli* (ATCC 8739), *Escherichia coli* I (clinical strain), and *Proteus mirabilis* (clinical strain) and the gram-positive bacteria *Staphylococcus aureus* (ATCC 25923), *Staphylococcus epidermidis* (clinical strain), and *Enterococcus faecalis* (ATCC 29212) were determined, and results are expressed as minimal inhibitory concentration (MIC) values in Table 4.

These bacterial strains are chosen for antibacterial activity testing because they are among the most important causes of serious community bacterial infections in humans [27].

The study provided evidence that all the honey samples manifested antibacterial activity against the studied strains, e.g., inhibited bacterial growth (Table 4). According to the following resistant potencies, *Escherichia coli* > *Escherichia coli* ATCC 8739 > *Enterococcus faecalis* > *Proteus mirabilis* > *Staphylococcus* *aureus* > *Staphylococcus* *epidermidis*. It was concluded that the examined honey samples demonstrated better inhibitory effects on gram-positive bacteria. The same effect of honey was previously observed by Farkasovska et al. [47] and Szweda [27]. The difference in sensitivity between gram-positive and gram-negative bacteria on the antibacterial activity of honey stems from the difference in the composition of their cell walls. Compared to gram-negative bacteria, gram-positive bacteria have no outer membrane to protect the peptidoglycan layer, which makes it easier for antimicrobial agents to penetrate and cause damage [48].

Linden honey sample 1 (from Fruška Gora) and phacelia honey samples 1 and 2 showed the strongest antibacterial activity. The average MIC values of 3.12% and 6.25% against gram-positive bacteria *Staphylococcus* *aureus* and *Staphylococcus* *epidermidis*, respectively, were determined for linden honey, while phacelia honey samples exhibited MIC values of 6.25% and 3.12% against *Staphylococcus* *aureus* and *Staphylococcus* *epidermidis*, respectively (Table 4). Junie et al. [49] also studied the antimicrobial activity of 10 different honey samples and established that the most sensitive bacteria to honey antibacterial activity were *Staphylococcus* *aureus* and *Staphylococcus* *epidermidis*. Nemo and Bacha [50] also found *Staphylococcus* *aureus* to be the most susceptible among investigated bacteria against honey. Contrary to these findings, Đogo Mračević et al. [17] revealed that honey from different regions of Serbia exhibited superior antibacterial potential against *Escherichia* *coli* compared to *Staphylococcus* *aureus*. The reason for the existence of such differences can be attributed to floral origin diversity and countries of origin.

The antibacterial potential of linden honey could be related to methyl syringate, which was found to be the most abundant component besides lindenin in linden honey [51] and known to act as an antibacterial agent [11].

Phacelia honey was previously established to exhibit the strongest antibacterial activity against *Staphylococcus* *aureus* among the investigated honey types, giving and MIC average value of 13.9%, while acacia and rapeseed honeys were weaker [52].

### 3.4. Antiproliferative Activity

Honey samples were evaluated in a broad range of concentrations from 0.15–50 mg/mL. The most active samples were linden honey sample 1 (IC_50_^MCF7^ = 7.46 ± 1.18 mg/mL and IC_50_^HeLa^ = 12.4 ± 2.00 mg/mL) and meadow sample 2 (IC_50_^MCF7^ = 12.0 ± 0.57 mg/mL, IC_50_^HeLa^ = 16.9 ± 1.54 mg/mL and IC_50_^HT−29^ = 23.7 ± 1.33 mg/mL) towards breast (MCF7), cervix (HeLa), and colon (HT-29) cancer cells. The most active samples, linden 1 and meadow 2 also affected the growth of MRC-5 cells derived from healthy lung tissue with IC_50_^MRC−5^ = 9.93 ± 0.68 mg/mL and IC_50_^MRC−5^ = 12.9 ± 0.34 mg/mL, respectively. Colon carcinoma cell line HT-29 was the least sensitive to the evaluated samples. Standard (glucose) had lower and uniform cell growth effect with IC_50_ values ranging from 33–40 mg/mL towards all evaluated cell lines, indicating that active components in samples other than sugars contributed to cell growth activity.

The antiproliferative activity of honey and honey-containing polyphenols on various cancer cell lines was recognised by Jaganathan and Mandalin [53]. Despite the statement that polyphenols have one of the crucial roles in the suppression of cancer cell growth [54], our results indicate that other mechanisms/compounds contribute to this phenomenon. Linden honey expressed the highest activity toward investigated cancer cell lines (Table 5) even though its TPC was much lower compared to basil honey (Table 3), whose activity in the suppression of cancel cell growth was not found (Table 5). These results can be explained by the fact that different polyphenols do not contribute equally to cell growth activity. Some phenolic acids like ellagic or gallic acid and the flavonoid kaempferol have exquisite antiproliferative activity (IC_50_ = 2 μg/mL), while the activity of ferulic and syringic acids and flavonoid rutin is mild [54].

Also, chestnut honey exhibited slight activity in the case of breast cancer cells with no effects on other examined lines (Table 5), although its TPC was relatively high (Table 3). Contrarily, chestnut honey from Anatolia was nominated as a powerful source of phenolic content, which is compatible with its cytotoxic affectivity towards breast cancer cell lines [55]. Therefore, more detailed polyphenolic profile investigation will be needed to correlate antiproliferative activities and polyphenol contents.

### 3.5. Principal Component Analysis

**Figure 1:**
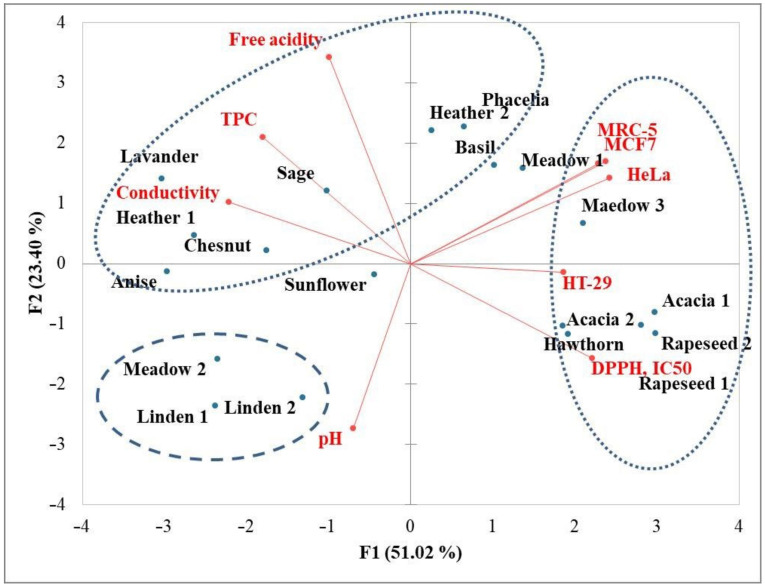
Principal component analysis (PCA) of physicochemical parameters and antioxidant and antiproliferative activities based on component correlations for the honey samples from the Western Balkans.

Principal component analysis (PCA) distinctively separated (PCA = 74.20%) samples with high antiproliferative activity (third quadrant) from those with low antiproliferative and low antioxidant activity (both samples of acacia and rapeseed honey, hawthorn and meadow sample 3) (Figure 1).

It is interesting that samples with high antiproliferative activity showed moderate antioxidant activity and possess moderate total phenolic content. A similar finding that antioxidant activity was not always correlated with the antiproliferative activity was reported in other studies [56,57].

## 4. Conclusions

The physicochemical characterisation and evaluation of antioxidative, antibacterial and antiproliferative effects were conducted for nineteen samples of different honey types (acacia, linden, heather, sunflower, phacelia, basil, anise, sage, chestnut, hawthorn, lavender and meadow) collected from different locations in the Republic of Serbia, Kosovo and Bosnia and Herzegovina, and Northern Macedonia.

Regarding the examined physicochemical parameters (moisture, pH, electrical conductivity, free acidity and HMF), all the samples were compliant with the levels defined by EU regulations with the exception of the electrical conductivity of heather and chestnut honey, whose conductivity values were above 0.8 mS/cm.

The highest phenolic content was found in basil honey (101 ± 2.72 mg GAE/100 g) and the lowest was registered in rapeseed honey (11.5 ± 0.70 mg GAE/100 g). DPPH scavenging activity was the highest for lavender honey (IC_50_ = 88.2 ± 2.11 mg/mL) and the lowest for rapeseed honey (IC_50_ = 646 ± 8.72 mg/mL).

Linden honey from Fruška Gora (MIC values of 3.12% and 6.25% against *Staphylococcus* *aureus* and *Staphylococcus* *epidermidis*, respectively) and phacelia honey (MIC values of 6.25% and 3.12% against *Staphylococcus* *aureus* and *Staphylococcus* *epidermidis*, respectively) showed the strongest antibacterial activity among the examined bacterial strains.

The highest antiproliferative activity was obtained by linden honey from Fruška Gora (IC_50_^MCF7^ = 7.46 ± 1.18 mg/mL and IC_50_^HeLa^ =12.4 ± 2.00 mg/mL) and meadow honey sample 2 (IC_50_^MCF7^ = 12.0 ± 0.57 mg/mL, IC_50_^HeLa^ = 16.9 ± 1.54 mg/mL and IC_50_^HT−29^ = 23.7 ± 1.33 mg/mL) towards breast (MCF7), cervix (HeLa) and colon (HT-29) cancer cells. The majority of honey samples had the most pronounced and selective action towards breast cancer cells (MCF7), with comparable or lower activity towards cells derived from healthy tissue (MRC-5). Active components other than sugars contributed to cell growth activity.

The investigated honey samples varied in antioxidative, antibacterial, and antiproliferative properties due to botanical and geographical variations. The influence of geographical origin is especially noticeable in the case of linden honey (samples 1 and 2) and meadow honey (samples 2 and 3). The flora of the region from which the honey samples were collected significantly contributed to the increased potency of the linden honey from Fruška Gora (sample 1) and the meadow honey from Kosovo (sample 2) in terms of the investigated parameters than the same honey type from another region.

## Figures and Tables

**Table 1:** Type of honey sample, location, and dominant/secondary pollen.

Honey Type	Location	Region	Dominant/Secondary Pollen
Acacia 1	Alibunar	Bačka, Vojvodina	*Robinia pseudoacacia*
Acacia 2	Fruška Gora	Srem, Vojvodina	*Robinia pseudoacacia*
Linden 1	Fruška Gora	Srem, Vojvodina	*Tilia* sp.
Linden 2	Đerdap	northeastern Serbia	*Tilia* sp.
Heather 1	Popovo Polje	eastern Herzegovina	*Calluna vulgaris*
Heather 2	Ljubuški	Bosnia and Herzegovina	*Calluna vulgaris*
Rapeseed 1	Sečanj	Banat, Vojvodina	*Brassica napus*
Rapeseed 2	Tovariševo	Bačka, Vojvodina	*Brassica napus*
Sunflower	Apatin	Bačka, Vojvodina	*Helianthus annuus*
Phacelia	Šajkaš	Bačka, Vojvodina	*Phacelia tanacetifolia*
Basil	Novi Kneževac	Banat, Vojvodina	*Stachys annua*
Anise	Probištip	Northern Macedonia	*Pinpinella* sp.
Sage	Ljubuški	Bosnia and Herzegovina	*Salvia officinalis*
Chestnut	Cazin	Bosnia and Herzegovina	*Castanea* sp.
Hawthorn	Cer	northwestern Serbia	*Crataegus monogyna*
Lavender	Fruška Gora	Srem, Vojvodina	*Lavandula stoechas*
Meadow 1	Trebinje	Republic of Srpska, BH	
Meadow 2	Leposavić	Kosovo	
Meadow 3	Plandište	Banat, Vojvodina	

**Table 2:** Physicochemical parameters of different types of honey.

Honey Type	Moisture (%)	pH	Electrical Conductivity (μS/cm)	Free Acidity (meq/kg)	HMF (mg/kg)
Acacia 1	17.3 ± 0.10 ^c^	3.90 ± 0.01 ^gh^	136 ± 3.51 ^no^	13.8 ± 0.26 ^h^	4.02 ± 0.04 ^fg^
Acacia 2	16.4 ± 0.35 ^ef^	4.51 ± 0.02 ^bc^	114 ± 2.65 ^o^	16.3 ± 0.30 ^g^	3.23 ± 0.11 ^gh^
Linden 1	15.8 ± 0.06 ^hi^	4.62 ± 0.02 ^ab^	488 ± 10.5 ^h^	16.1 ± 0.21 ^g^	7.04 ± 0.98 ^c^
Linden 2	17.1 ± 0.23 ^cd^	4.72 ± 0.02 ^a^	608 ± 2.08 ^f^	14.5 ± 0.12 ^gh^	5.46 ± 0.21 ^d^
Heather 1	16.7 ± 0.21 ^de^	4.32 ± 0.11 ^d^	834 ± 8.39 ^d^	39.2 ± 2.19 ^a^	5.41 ± 0.17 ^de^
Heather 2	15.6 ± 0.21 ^i^	3.36 ± 0.03 ^l^	809 ± 7.77 ^d^	30.7 ± 1.30 ^bc^	3.27 ± 0.06 ^gh^
Rapeseed 1	19.4 ± 0.15 ^a^	4.01 ± 0.04 ^efg^	224 ± 3.79 ^l^	21.3 ± 0.68 ^f^	2.42 ± 0.09 ^hi^
Rapeseed 2	18.4 ± 0.26 ^b^	4.10 ± 0.03 ^e^	191 ± 7.77 ^m^	16.3 ± 0.46 ^g^	7.15 ± 1.00 ^c^
Sunflower	17.0 ± 0.53 ^cd^	3.38 ± 0.22 ^kl^	366 ± 13.1 ^j^	28.9 ± 1.78 ^cde^	9.41 ± 0.70 ^a^
Phacelia	15.0 ± 0.21 ^j^	3.66 ± 0.06 ^i^	295 ± 4.73 ^k^	37.2 ± 2.00 ^a^	1.89 ± 0.30 ^i^
Basil	16.0 ± 0.20 ^gh^	3.84 ± 0.03 ^h^	413 ± 7.09 ^i^	27.9 ±0.35 ^de^	3.14 ± 0.13 ^gh^
Anise	16.4 ± 0.30 ^efg^	4.34 ± 0.04 ^d^	879 ± 8.74 ^c^	31.2 ± 0.89 ^b^	5.58 ± 0.86 ^d^
Sage	15.1 ± 0.21 ^j^	4.07 ± 0.06 ^ef^	557 ± 8.89 ^g^	37.7 ± 1.94 ^a^	4.94 ± 0.84 ^def^
Chestnut	16.5 ± 0.25 ^ef^	4.54 ± 0.11 ^b^	1251 ± 41.7 ^a^	27.1 ± 0.26 ^e^	4.43 ± 0.42 ^ef^
Hawthorn	19.1 ± 0.31 ^a^	4.41 ± 0.04 ^cd^	163 ± 12.5 ^mn^	20.3 ± 0.36 ^f^	8.25 ± 0.42 ^b^
Lavender	15.8 ± 0.26 ^hi^	3.65 ± 0.04 ^i^	1040 ± 54.1 ^b^	38.9 ± 1.63 ^a^	7.73 ± 1.01 ^bc^
Meadow 1	14.9 ± 0.15 ^j^	3.59 ± 0.09 ^ij^	353 ± 12.1 ^j^	37.8 ± 2.76 ^a^	1.85 ± 0.12 ^i^
Meadow 2	17.4 ± 0.40 ^c^	3.95 ± 0.04 ^fgh^	715 ± 11.9 ^e^	20.1 ± 0.70 ^f^	3.41 ± 0.34 ^gh^
Meadow 3	16.3 ± 0.12 ^fg^	3.50 ± 0.15 ^jk^	470 ± 23.7 ^h^	29.5 ± 0.75 ^bcd^	1.81 ± 0.58 ^i^

Means in the same column with different superscript are statistically different (*p* ≤ 0.05).

**Table 3:** Phenolic content and DPPH radical scavenging activity of different types of honey.

Honey Type	Polyphenols (mg GAE/100 g)	DPPH, IC_50_ (mg/mL)
Acacia 1	14.4 ± 0.49 ^kl^	442 ± 19.3 ^b^
Acacia 2	13.5 ± 0.35 ^lm^	388 ± 10.1 ^d^
Linden 1	67.3 ± 2.57 ^f^	115 ± 9.00 ^l^
Linden 2	53.7 ± 3.37 ^g^	223 ± 12.3 ^g^
Heather 1	79.3 ± 1.01 ^e^	137 ± 7.55 ^k^
Heather 2	84.0 ± 3.83 ^g^	156 ± 10.3 ^j^
Rapeseed 1	11.5 ± 0.70 ^m^	646 ± 8.72 ^a^
Rapeseed 2	11.9 ±0.25 ^lm^	640 ± 22.5 ^a^
Sunflower	27.5 ± 0.50 ^i^	324 ± 5.51 ^e^
Phacelia	89.7 ±0.99 ^c^	175 ± 4.36 ^hi^
Basil	101 ± 2.72 ^a^	162 ± 5.29 ^ij^
Anise	98.7 ± 0.90 ^a^	186 ± 4.04 ^h^
Sage	90.1 ± 1.76 ^c^	184 ± 9.29 ^h^
Chestnut	88.8 ± 1.55 ^c^	193 ± 11.0 ^h^
Hawthorn	36.7 ± 1.34 ^h^	415 ± 14.0 ^c^
Lavender	95.6 ± 1.06 ^b^	88.2 ± 2.11 ^m^
Meadow 1	24.5 ± 1.01 ^j^	266 ± 6.03 ^f^
Meadow 2	26.5 ±0.76 ^ij^	224 ± 6.11 ^g^
Meadow 3	16.8 ±0.50 ^k^	428 ± 14.2 ^bc^

Means in the same column with different superscript are statistically different (*p* ≤ 0.05). GAE—gallic acid equivalent

**Table 4:** Minimum inhibitory concentrations (MIC, %) of different types of honey against tested strains of *Escherichia coli* (ATCC 8739), *Escherichia coli*, *Proteus mirabilis*, *Staphylococcus aureus* (ATCC 25923), *Staphylococcus epidermidis*, and *Enterococcus faecalis* (ATCC 29212).

Honey Type	MIC % against Different Strains of Bacteria					
	*Escherichia* *Coli* (ATCC 8739)	*Escherichia* *coli*	*Proteus Mirabilis*	*Staphylococcus Aureus* (ATCC 25923)	*Staphylococcus Epidermidis*	*Enterococcus Faecalis* (ATCC 29212)
Acacia 1	25	25	25	25	12.5	>25
Acacia 2	25	25	25	12.5	25	>25
Linden 1	25	25	12.5	3.12	6.25	25
Linden 2	25	25	12.5	12.5	12.5	25
Heather 1	25	25	12.5	6.25	12.5	25
Heather 2	25	25	25	12.5	12.5	25
Rapeseed 1	>25	>25	>25	>25	>25	>25
Rapeseed 2	>25	>25	25	25	25	>25
Sunflower	25	25	12.5	12.5	12.5	25
Phacelia	12.5	25	12.5	6.25	3.12	25
Basil	25	25	12.5	12.5	12.5	25
Anise	25	25	12.5	6.25	12.5	25
Sage	25	25	25	12.5	12.5	25
Chestnut	25	25	25	12.5	12.5	25
Hawthorn	>25	>25	25	25	25	25
Lavender	25	25	25	12.5	12.5	25
Meadow 1	25	25	25	12.5	12.5	25
Meadow 2	25	25	12.5	6.25	6.25	25
Meadow 3	25	25	25	12.5	12.5	25

The determination of MIC was performed in triplicate.

**Table 5:** Effects of honey samples on the growth of selected human cell lines.

Honey Type	IC_50_ (mg/mL) *			
	HeLa	MCF7	HT-29	MRC-5
Acacia 1	>50 ^g^	>50 ^f^	>50 ^e^	>50 ^f^
Acacia 2	>50 ^g^	>50 ^f^	30.4 ± 5.95 ^b^	>50 ^f^
Linden 1	12.4 ± 2.00 ^a^	7.46 ± 1.18 ^a^	43.3 ± 3.68 ^d^	9.93 ± 0.68 ^a^
Linden 2	25.9 ± 1.68 ^d^	17.2 ± 5.24 ^c^	41.5 ± 2.36 ^d^	19.8 ± 1.38 ^b^
Heather 1	24.0 ± 1.54 ^d^	18.5 ± 0.52 ^c^	30.0 ± 2.49 ^b^	25.2 ± 0.41 ^c^
Heather 2	>50 ^g^	40.4 ± 8.75 ^e^	42.9 ± 1.36 ^d^	>50 ^f^
Rapeseed 1	>50 ^g^	35.8 ± 10.1 ^e^	>50 ^e^	>50 ^f^
Rapeseed 2	>50 ^g^	>50 ^f^	42.3 ± 3.58 ^d^	>50 ^f^
Sunflower	24.8 ± 0.28 ^d^	27.7 ± 1.95 ^d^	35.6 ± 2.68 ^c^	20.7 ± 2.56 ^b^
Phacelia	>50 ^g^	>50 ^f^	36.7 ± 2.16 ^c^	>50 ^f^
Basil	>50 ^g^	>50 ^f^	>50 ^e^	>50 ^f^
Anise	21.0 ± 0.56 ^c^	14.7 ± 2.42 ^b^	28.4 ± 6.64 ^b^	21.9 ± 2.58 ^b^
Sage	35.2 ± 7.50 ^e^	26.7 ± 0.20 ^d^	34.3 ± 0.82 ^c^	45.0 ± 2.31 ^e^
Chestnut	40.3 ± 1.71 ^f^	25.6 ± 0.27 ^d^	37.2 ± 0.86 ^c^	34.8 ± 0.89 ^d^
Hawthorn	>50 ^g^	35.1 ± 11.7 ^e^	49.0 ± 0.06 ^e^	44.7 ± 2.28 ^e^
Lavender	20.8 ± 2.66 ^c^	24.5 ± 2.52 ^d^	32.0 ± 4.93 ^b^	17.3 ± 0.53 ^b^
Meadow 1	>50 ^g^	49.0 ± 0.71 ^f^	37.4 ± 1.30 ^c^	>50 ^f^
Meadow 2	16.9 ± 1.54 ^b^	12.0 ± 0.57 ^b^	23.7 ± 1.33 ^a^	12.9 ± 0.34 ^a^
Meadow 3	>50 ^g^	>50 ^f^	>50 ^e^	40.3 ± 8.93 ^e^
Standard				
Glucose	40.0 ± 3.02 ^f^	33.2 ± 5.57 ^e^	34.5 ± 0.44 ^c^	39.8 ± 1.07 ^e^

* Values represent means ± SD of four (*n* = 4; test samples and standard) or eight (*n* = 8, controls); measurements obtained in 0.15–50 mg/mL concentration range. Means within each column with different letters differ significantly (*p* ≤ 0.05). HeLa—HeLa human cervical carcinoma cell line; MCF7—MCF7 human breast adenocarcinoma cell line; HT-29—HT-29 human colorectal adenocarcinoma cell line; MRC-5—MRC-5 human lung cell line.

## Data Availability

Data is contained within the article.
